# Reducing the need for repeating urine drug testing with the gray zone determined by the measurement uncertainty

**DOI:** 10.5937/jomb0-41777

**Published:** 2023-10-27

**Authors:** Kagan Huysal, Yasemin Üstündağ, Hatice Azra Çağlak, Yeşil Meryem Rümeysa

**Affiliations:** 1 University of Health Sciences, Bursa Yüksek Ihtisas Training and Research Hospital, Department of Clinical Biochemistry, Bursa, Turkey

**Keywords:** cut-off, grey zone, measurement uncertainty, amphetamine, cannabinoid, cocaine, granica, siva zona, merna nesigurnost, amfetamin, kanabinoid, kokain

## Abstract

**Background:**

On an initial urine screening test for illegal substances, if the concentration of a substance is at or above the determined legislative threshold, it is reported as positive. Repeating testing with the same sample to verify it before reporting is a common practice in clinical laboratories. This study aimed to determine whether measurement uncertainty (MU) results can be used to detect a grey zone to reduce repeat testing.

**Methods:**

A retrospective study was conducted using data from the laboratory information system between January 1, 2020, and July 1, 2022. Samples studied twice within one hour before reporting for the same urine sample were analyzed. The MU values for urinary amphetamine, cannabinoid, cocaine, and opioid parameters were calculated using ADVIA Chemistry reagents on a Siemens ADVIA 1800 chemical analyzer. The grey zone was defined as the cut-off value ± MU.

## Abstract (sr)

**Uvod:**

Ako je koncentracija nedozvoljene supstance na ili iznad utvrđenog zakonskog praga na početnom testu skrininga urina na nedozvoljene supstance, takvi rezultati se prijavljuju kao pozitivni. Ponavljanje testiranja sa istim uzorkom radi verifikacije pre prijavljivanja je uobičajena praksa u kliničkim laboratorijama. Ova studija je imala za cilj da utvrdi da li se rezultati merne nesigurnosti (MU) mogu koristiti za otkrivanje sive zone kako bi se smanjila ponovljena testiranja.

**Metode:**

Sprovedena je retrospektivna studija korišćenjem podataka iz laboratorijskog informacionog sistema između 1. januara 2020. i 1. jula 2022. Analizirani su isti uzorci koji su dvaput proučavani u roku od jednog sata pre nego što su prijavljeni. Vrednosti MU za parametre amfetamina, kanabinoida, kokaina i opioida u urinu su izračunate korišćenjem ADVIA Chemistry reagensa na Siemens ADVIA 1800 hemijskom analizatoru. Siva zona je definisana kao granična vrednost ± MU.

**Rezultati:**

Kod 31,839 pacijenata je izvršeno ponovljeno uzorkovanje urina, i ponovo je urađeno 319 testova na amfetamine, 198 na kanabinoide, 112 na kokain, kao i 125 testova na opijate (16-65 godina). Deset rezultata testova na amfetamine, 12 na kanabinoide i 2 rezultata testa na kokain su se promenili sa pozitivnih na negativne ili sa negativnih na pozitivne na ponovnom testiranju, sve u okviru nivoa sive zone definisane MU vrednošću. Prosečna odstupanja između rezultata ponovljenih testova bila su -7,64 (95% CI: -13,71 do -1,57) ng/mL za amfetamin i 1,16 (95% CI: 0,31 do 2,01) ng/mL za rezultate kanabinoida.

**Zaključak:**

Ponavljanje samo onih vrednosti unutar sive zone za svaku nedozvoljenu supstancu moče biti korisno za smanjenje nepotrebnih duplih merenja.

## Introduction

Illicit substance testing performed on biological samples is an important tool for assessing and monitoring the risk of addiction and abuse. Misinterpreting test results can lead to legally calculable consequences [1] [2]. Urine is the primary matrix for the detection of substance use due to the ease of sample collection compared to blood collection and an extended drug detection period [1] [2].

A two-step analysis procedure, consisting of initial screening and confirmatory testing steps, is the common approach to urine drug testing [1] [2]. Enzyme immunoassay tests, frequently used in the screening step, provide rapid results with low-cost test kits and routine chemistry instruments [3].

A cut-off level is established on an initial urine drug test; if the concentration is at or above the determined legislative threshold, the substance is reported as positive [1] [2] [3] [4].

Depending on the Gaussian distribution of the results at the threshold concentration, there is a risk of classifying positive results as negative and vice versa by the immunoassay methods [5]. Furthermore, false-positive or rarely false-negative test results can be reported due to the interaction of antibodies with molecules with similar structures [6].

The area around the cut-off value, known as the "grey zone," is defined to express the uncertainty of results [7] [8]. Using a grey zone to classify screening immunoassay test results reduces the risk of misclassification; however, this is not systematically needed [7]. Furthermore, its use is not required by the ISO 15189 guideline, which specifies the requirements for the accreditation of medical laboratories [9].

An illicit urine substance screening test result is interpreted as the actual value, and a decision is made according to this result [4]. To make a clinical decision, a qualitative test is usually repeated with the same method and/or confirmatory testing is performed to ensure accuracy [10] [11].

This study aims to determine whether the measurement uncertainty (MU) results of urinary amphetamine, cannabinoid, cocaine and opioid parameters studied using ADVIA Chemistry reagents (Siemens Healthcare Diagnostic Inc., Germany) on a Siemens ADVIA 1800 chemical analyzer for urinary illegal substance screening can be used to detect a grey zone to reduce incidences of repeat testing.

## Materials and methods

This study was approved by the Institutional Ethics Committee (2011-KAEK-252022/08-08). The study was conducted at the Alcohol and Substance Research, Treatment and Training Center of the Bursa Yuksek Ihtisas Research Hospital. Data from the Hospital Information Management and Laboratory Information System between January 1, 2020, and July 1, 2022, were retrospectively analyzed. Siemens amphetamine (ADVIA Chemistry, AMPH_2), cannabinoid (ADVIA Chemistry Cannabinoid_2, THC_2), cocaine (ADVIA Chemistry Cocaine Metabolite_2 COCA_2), and opiate (ADVIA Chemistry Opiate_2, OP_2) kits were measured with the Siemens ADVIA 1800 chemical analyzer using the enzyme multiplied immunoassay technique, which is based on competition between substance and enzyme glucose-6-phosphate dehydrogenase for antibody binding sites. Among the urine samples analyzed for illicit substances, those studied twice within one hour for confirmation were evaluated.

Two levels of commercially available internal quality control materials (Detectabuse Urine Liquid QC; Control set low/high, Lot HA10125, HA10175; Lot 904125, 904175; Biochemical Diagnostics nc., USA) were assayed in duplicate twice daily during the study period. The manufacturer's package inserts were followed.

For quality assurance purposes, our laboratory participates in an external quality control program (Oneworld Accuracy, Canada).

The MU for each urine illicit drug test was calculated as previously described [12]. The established urine illicit drug tests cut-off concentrations are amphetamines 500 ng/ml, cannabinoids 50 ng/ml, cocaine 150 ng/ml, and opioids 2.000 ng/ml.

This study defined the grey zone as the cut-off value ± MU of the analytical region.

### Statistics

Bland–Altman plots were evaluated using MedCalc® Statistical Software version 20.121 (MedCalc Software Ltd, Ostend, Belgium).

## Results

**Figure 1:**
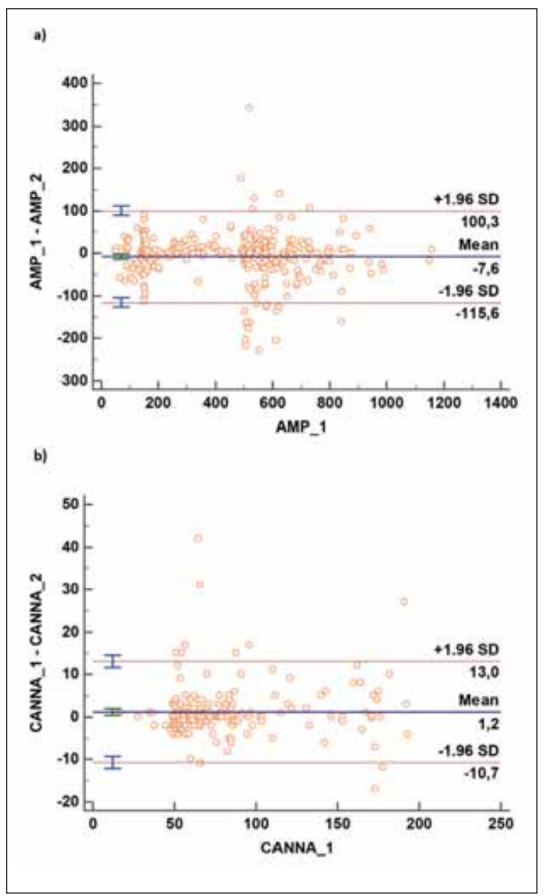
The difference plot between the two test runs of individual specimens plotted against the mean results of the 2 test runs: a) Amphetamine and b) Cannabinoid. The outer solid lines are the upper and lower limits of agreement.

During the study period, a panel of urine amphetamines, cocaine, cannabinoids, and opiate levels were simultaneously measured for 31.839 patients (16-65 years), of which 319 amphetamine, 198 cannabinoids, 112 cocaine, and 125 opiate tests were repeated. Mean biases between the repeat test results were -7.64 (95% CI: −13.71 to -1.57) ng/ml for the amphetamine and 1.16 (95% CI: 0.31 to 2.01) ng/mL for the cannabinoid results (Figure 1).

**Figure 2:**
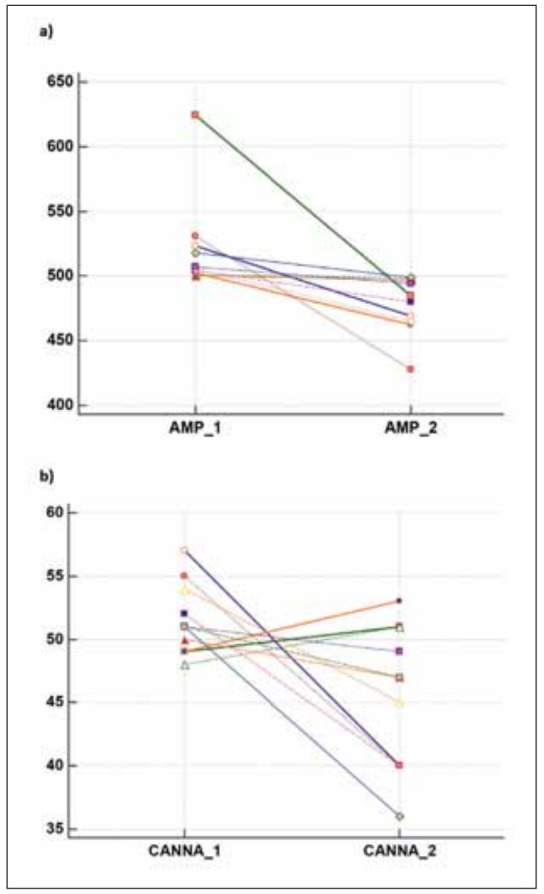
Dot plot illustrating the comparison of repeated test results: a) Amphetamine, b) Cannabinoid.

The results for 11 amphetamines, 12 cannabinoids, 2 urinary cocaine tests close to the cut-off levels changed from positive to negative or negative to positive in the retests (Figure 2). There was no change from positive to negative or negative to positive in the urine opiate test results.

MU values (k=3, level of confidence >99%) were calculated as 30.9% for amphetamine, 17.2% for cannabinoids, 20.4% for cocaine, 10.5% for opiates, and 21% for synthetic cannabinoids. Based on the MU results, the grey zones were determined as 345-655 ng/ml for amphetamine, 43-59 ng/ml for cannabinoids, 120-180 ng/ml for cocaine, and 1.790-2.210 ng/mL for opiates.

The reported false-positive/false-negative test result rate was 0.018%. All values that changed from negative to positive and from positive to negative as a result of test repetitions were within the grey zone range calculated with the MU. These results were within the following ranges: 407-624 ng/mL for amphetamine, 40-55 ng/mL for cannabinoids, and 150-155 ng/mL for cocaine. Because we do not have a rule for retesting, we repeated a total of 754 tests (319 amphetamine, 198 cannabinoid, 112 cocaine, and 125 opiate). Only 211 tests (127 amphetamine, 67 cannabinoid, 10 cocaine, and 7 opiate tests) needed to be retested, as the results were within the calculated grey zone.

## Discussion

During the study period, 31.839 patients' urinary samples were analyzed, and 754 tests were repeated with the same samples. Only 26 results changed: 12 amphetamine, 12 cannabinoid, and 2 cocaine results. Although the number of patients whose results changed is low, the impact of a falsepositive or false-negative result is critical, considering legal implications [12] [13].

All values that changed from negative to positive or vice versa were within the grey zone limits calculated with the MU method. The grey zone concept is widely used in biochemical parameters. However, the guidelines do not refer to determining the grey zone in reporting illicit substances [14]. The current literature suggests using the grey zone approach to immunologically screen blood donors for infectious diseases and pharmacological tests (such as serum growth hormone stimulation tests) [5] [15]. Coste et al. reported that using the grey zone approach in conjunction with the Bland-Altman method should increase reliability [16].

Several guidelines and recent research have proposed replacing traditional immunoassay methods in urine illicit drug testing with more accurate tests, such as liquid chromatography-tandem mass spectrometry (LC-MS/MS) [15] [17] [18]. However, immunoassays are preferred in routine use for drug testing programs, as they provide rapid results. LC-MS/MS is not available at several institutions, and the cost to the laboratories is considered too expensive by many governments [18].

It is essential to be aware of the screening performance characteristics of immunoassay drug tests to avoid any misinterpretation [19]. Grey zone detection can be a reliable tool to reduce the workload of costly confirmation methods, such as retesting using immunoassay systems and LC-MS/MS tests.

The logic of the retest rules is that the repeat test result can correct an analytical error that is revealed by the first test. However, with technological progress, analytical errors are becoming less and less common [20] [21]. If the performance of the analyzers has been checked beforehand, repeat testing to verify their accuracy increases laboratory costs [22] [20]. Studies have shown that automated repeat test results are similar to the original result, wasting resources without significantly preventing analytical errors, and are therefore unnecessary [22] [23] [24].

During our study period, 319 amphetamine, 198 cannabinoid, 112 cocaine, and 125 opiate tests were repeated. Repeat testing has been shown to be unnecessary in most cases if repetitions are made according to the calculated MU value. In this study, only 211 tests needed to be repeated instead of 754. Each laboratory is advised to create its own protocol for repeat testing based on its own practice, significantly reducing cost [21].

One possible limitation of this study is further examining the test repetition rates in our laboratory. Currently, there is no rule for illicit drug repeat testing, which is usually subjectively required by the laboratory technician.

In conclusion, repeating only values within the grey area for each illicit substance may be useful to reduce unnecessary duplicate measurements. In the classification of screening results, only values within the grey zone indicate a retest and confirmatory analyses should be conducted in the case of positivity.

## Dodatak

### Acknowledgements

Nothing to declare.

### Conflict of interest statement

All the authors declare that they have no conflict of interest in this work.

### List of abbreviations

LC-MS, liquid chromatography-mass spectrometry; 

MU, measurement uncertainty.
